# Media education and educational commons for youth civic engagement. A case study from the Horizon 2020 project SMOOTH

**DOI:** 10.3389/fsoc.2022.1108229

**Published:** 2023-01-12

**Authors:** Gianna Cappello, Marianna Siino

**Affiliations:** ^1^Department of Psychology, Educational Science, and Human Movement, University of Palermo, Palermo, Italy; ^2^Department of Cultures and Society, University of Palermo, Palermo, Italy

**Keywords:** media education, media literacy, educational commons, participatory culture, action research, young people, civic engagement

## Abstract

This study presents the preliminary findings of the first round of implementation of a case study included in the Horizon 2020 project SMOOTH. The project's main objective is to introduce and study the emergent paradigm of the educational commons as an alternative system of values and actions for promoting intercultural and intergenerational dialogue and establishing spaces of democratic citizenship that support the development of local communities. Our case study adopts this paradigm with insights derived from the field of media education. Hence, our research questions were as follows: (a) How do young people collectively experience and build the educational commons? (b) How do participants (youth and adults) in educational commons experience peer governance and how do they handle and resolve conflicts? (c) How does the co-creation of a photo-blog as a shared space of work help young people discover and develop a "civic intentionality" in the (digital) public sphere? (d) What are the effects of applying a commons' logic to address inequalities and achieve social inclusion of young people from vulnerable social groups? Fieldwork, framed in an ethnographic and action-research approach, was developed by examining the three dimensions of the notion of educational commons (commoners, commoning practices, and community). Although data collection and analysis are still in progress, our preliminary results allow us to draw some initial reflections on what worked well in the first round and what could hinder the achievement of the project's objectives. We also propose hypotheses for re-designing the second round to overcome the weaknesses that emerged during the first experimental phase and foster its strengths.

## 1. Introduction

The motivation for this study arose from the need to reconsider education as a shared resource and to examine the importance of experimenting with, monitoring, and evaluating (digital) co-creation practices that may engage and empower young people within their communities.

This perspective views teachers, educators, young people, and families as commoners of a community that has no pre-established borders other than those inspired by a collective process of production and governance of a common good—education—which they protect and value as a key promoter of social justice and democracy. The emergent paradigm of educational commons represents an alternative and an example of resistance to the current neo-liberal processes of education closure and commodification. Like other commons, educational commons involve either sharing space or ways of being together in the world. However, they do not emerge spontaneously but are instead the challenging product of complex and arduous commoning processes based on a peer-governance logic through which people co-decide and set mutual limits by co-establishing specific rules for managing conflict and activities. In our specific case, online environments can act as a multiplier for the educational commons, as they can host decentralized, self-administered communities (coterminous with offline contexts) and offer new opportunities for producing and exchanging information, knowledge, and culture. These online communities can become fertile ground for participatory practices based on individual freedom, autonomous social collaboration, diversity, and co-production without hierarchal governance.

Given that empirical research and experimentation on educational commons is somewhat limited compared to other forms of commons, the project SMOOTH proposes an innovative action-research program on educational commons by engaging researchers, educators, children, youth, and the local community to reverse the inequalities faced by vulnerable social groups and engender social change. Partners from eight countries (Belgium, Estonia, Germany, Greece, Italy, Portugal, Spain, and Sweden) are involved in experimenting with over 50 case studies distributed in different educational settings (preschool, school, and youth clubs).

In this study, we present the preliminary findings of the first implementation round of one (out of two) Italian case studies where the paradigm of the educational commons is adopted in conjunction with insights derived from media education. Experimental activities intend to encourage young people to develop the skills, knowledge, ethical and critical frameworks, and self-confidence needed to express “civic intentionality” and be fully “engaged citizens” in the (digital) public sphere.

Fieldwork, framed in an ethnographic and action-research approach, was developed by examining the three dimensions of the notion of educational commons (commoners, common practices, and community).^1^ They were first defined theoretically and then operationalized into less abstract sub-dimensions to detect empirically useful information to assess the impact of the intervention. Therefore, the empirical basis of our analysis is made up of the textual data collected through the interviews and focus groups, the logbooks, the field notes, the observation grids, and the audiovisual documentation. Data collection is still underway, and the second set of experimental activities is expected to begin shortly. However, given the data collected thus far, we have attempted to (a) reconstruct the micro-context in which the case study activities took place, (b) comprehensively describe the relational dynamics, the processes, and the products using unobtrusive methods, and (c) assess the impact of the activities concerning the objective of developing “civic intentionality” and reducing educational inequalities through an educational-commons and media-education approach.

## 2. Experimenting with the educational commons

### 2.1. Commoning social life

The notion of educational commons draws from a large (and highly controversial) body of theories and practices of the commons, i.e., collective cultural or natural goods that are produced, governed, and shared in common, inspiring new modes of thinking and practicing democratic politics, economy, and culture, and furthering collective empowerment for responding to the political, socio-economic, and ecological crises of our times (Ostrom, 1990, 2012; Dardot and Laval, 2014). The commons can also be defined as a “malleable social relation” or a “social practice of commoning” by which a resource (i.e., a piece of land) is governed not by state or market entities but by a community of users through the institutions that it creates. Hence, the commons consist of three main components: (1) common resources/goods/spaces; (2) commoning practices; and (3) commoners who are implicated in the peer production and reproduction of the commons. In the world of peer governance—a *common verse*, as Bollier and Helfrich (2019) define it—individuals and groups have the same rights and obligations; they do not compete for control and power over others but are commoners participating in a collective process of governance. “At the heart of the practice of commoning lies the principle that the relation between the social group and that aspect of the environment being treated as a common shall be both collective and non-commodified-off-limits to the logic of market exchange and market valuations.” (Harvey, 2013, p. 73). Indeed, the commons are most often invoked as a direct challenge to neo-liberal hegemony and the “predatory formations” (Sassen, 2014) of global capitalism to commodify and therefore enclose what remains of the world's shared fund of natural and cultural wealth (Harvey, 2013).

The transformative potential of the commons is not a merely theoretical construct but a manifest reality, for example, urban commons and digital commons. Urban commons stem from citizens' groups in urban centers that increasingly reclaim public space and infrastructure, such as housing and energy supplies, and strive to run them as collectively self-managed resources for the common good. This allows them to experience other modes of civic participation and self-reliance beyond the state and the marketplace (Kioupkiolis, 2020). Urban commons differ from public spaces in cities, such as squares and infrastructure, which are subject to the power of the state and public administration. Public spaces and assets are transformed into commons when they are used and cared for by citizens who work together to protect and improve them for their mutual benefit (Bartoletti, 2013; Harvey, 2013; Dellenbaugh et al., 2015).

Similarly, with the advent of the Internet and, more recently, social media, there has been a shift in focus from the “commons of nature” to the commons of culture, information, and digital networks (Benkler and Nissenbaum, 2006; Bollier, 2008; Benkler, 2011; Bauwens et al., 2019; Dafermos, 2020). Digital environments have created decentralized, self-administered communities, offering new opportunities for producing and exchanging information, knowledge, and culture in diverse fields, from free software development to online encyclopedias, such as Wikipedia, to investigative journalism (such as citizen-journalism), video gaming, and fandom practices (such as fandubbing and fansubbing). These communities develop a “participatory culture” based on individual freedom, autonomous social collaboration, diversity, and co-production without hierarchal governance (Jenkins, 2006; Jenkins et al., 2013).

In short, the notion of the commons has expanded to include natural, ecological, social, and cultural goods (i.e., material and immaterial goods). All of them share a minimum socio-political semantic core, which can be identified in the following features: (1) the opposition of the concept of the commons to the dynamics of neoliberalism, (2) the re-composition of networks of cooperation within communities, and (3) the development of practices for participatory democracy (Coccoli, 2013).

### 2.2. Commoning education

Building on the praxis of the commons, the emerging paradigm of educational commons represents a possible alternative value and action system to reconfigure education and strengthen its contribution to fostering social justice and democracy (Pechtelidis and Kioupkiolis, 2020). It implies that learning, knowledge transmission, and acquisition processes are co-constructed and co-governed by the educational community—teachers, educators, students, and their families—in terms of participation, openness, and diversity. Teachers and educators relinquish their position as masters “transmitting” a fixed, authoritative tradition and instead support children in becoming “commoners,” i.e., self-directing, creative individuals who, while drawing from inherited cultural heritage and knowledge, also embark on their own innovative explorations and redefinitions. Educational commons, however, do not emerge spontaneously but are instead the challenging product of commoning processes based on a peer-governance logic through which people co-decide and set mutual limits by co-establishing specific rules and co-managing conflicts.

As with other commons, educational commons are both *spaces* to be together and *ways* of being together in the world (Korsgaard, 2019). They constantly struggle against enclosing pressures, which, in the case of school education, “take the form of privatization as a means of transforming K-12 and higher educational institutions and processes into potential investment opportunities and sites for profit extraction” (Means et al., 2017, p. 5). Enclosing pressures on education also come from the technocratic managerial logic associated with neo-liberalism (Rizvi and Lingard, 2009) and its ideological rhetoric about human capitalization, which traps educational value within an economy-driven schema that transforms people into “capital stocks” for the labor market (Lazzarato, 2012).

Empirical research and experimentation on education commons are limited in comparison to other forms of commons. There are some schools and communities (Means et al., 2017; Pechtelidis, 2018; Pechtelidis and Kioupkiolis, 2020) where a process of commoning education has been developed through the construction of alternative spaces for learning and participation where democracy, self-governance, social justice, and equality are promoted. In the educational commons, participants (adults and children) engage in alternative social relations, which foster new forms of subjectification (Biesta, 2011) and “citizenship.” Two concrete examples come from Greece: the Little Tree community in Thessaloniki (Pechtelidis and Kioupkiolis, 2020) and the Sprogs community in Volos (Pechtelidis, 2018), both formed by a group of preschool children, parents/guardians, and educators or pedagogues. The assembly plays a central role in the functioning of these communities. It is the place and the moment when these three groups make decisions on various issues that concern them and their community.

The commons' logic can also be developed in public schools, despite the strict requirements of official curricula and the institutional rules that usually regulate schools' everyday life and arrange people's bodies, space, and time. The preschools of the municipality of the Italian city of Reggio Emilia, inspired by Loris Malaguzzi's pedagogy, and a network of public schools in Sweden following this approach are excellent examples of commoning the public educational system (Moss, 2019). The educational commons approach has also been adopted in adult education schools in Barcelona (Aroca, 1999; Flecha, 2000) and by several groups of parents for childcare (Garganté, 2017). Aroca (1999) describes, for example, the philosophy and activities of La Verneda-Sant Martí, a school for adult education, where students (who call themselves participants), teachers, volunteers, and community members participate in the school's decision-making process. Similarly, Flecha (2000) narrates how the literary reading circles of this school allowed (migrant) adults to progress from the point where they never read books to the point where they enjoyed reading together the literary works of authors such as Lorca, Kafka, Dostoyevsky, and Joyce.

These experiences suggest the potential of the educational commons paradigm for transforming education (and society as a whole) through greater participation of citizens and local communities in developing viable policies and practices to overcome more utilitarian, individualistic, and neo-liberal approaches and build more democratic educational systems (UNESCO, 2015). Indeed, as Apple and Beane (2007) suggest, to strengthen democratic institutions, collective action needs to be generated from the bottom, from groups that provide the driving force for change. This is even more urgent, especially if we consider the crisis of welfare states in many countries worldwide and the ongoing privatization and commodification of education.

### 2.3. The Horizon 2020 research project SMOOTH “educational commons and active social inclusion”

The project SMOOTH, running from 2021 to 2024, proposes an innovative action-research program engaging educators, children, and youth to reverse the inequalities faced by vulnerable social groups and engender social change. It aims to introduce and study the emergent paradigm of the educational commons as an alternative value and action system to reinforce intercultural and intergenerational dialog, establishing spaces of democratic citizenship that support local community development. Adopting an action research approach is crucial for achieving these objectives. As we know, its fundamental epistemological and methodological perspective rests on a “self-reflective spiral,” involving an iterative process of planning, implementation, observation, and critical thinking for new planning. In addition, action research is participatory, as it allows a collective process of generating and reproducing the knowledge needed to transform reality, reducing the gap between research and practice. As such, it implies that researchers and practitioners change their behaviors during the design, implementation, and evaluation of educational activities (Somekh, 2006). The action itself provides the raw research material since the aim is not merely to collect data on reality but also to transform it.

In the SMOOTH project, universities, research labs, municipalities, NGOs, museums, and youth organizations from eight different countries (Belgium, Estonia, Germany, Greece, Italy, Portugal, Spain, and Sweden) are working together, experimenting with over 50 case studies distributed in different educational settings (preschool, school, and youth clubs). Each case study involves at least a third party (a school, a youth club, and so on) and is designed to be replicated over two rounds. Data will be extracted and analyzed between the first and second rounds, and if empirical evidence requires, the second round will be modified. Research questions to guide experimentation include:

- Are there similar tendencies in the various educational commons of the project?
- Can education be organized effectively using educational commons models?
- What are the effects of applying the commons' logic to address inequality and social inclusion for children and youth of vulnerable social groups?
- Can commons-based peer education contribute to developing peer production?
- How do children and youth collectively experience and build the commons in formal, non-formal, and informal educational settings?
- How do the use and co-development of digital devices and online environments enable children and youth to discover and develop their own priorities and improve active inclusion?
- Do gender (or other kinds of) differences affect how children and youth engage in educational commons?
- How do participants (children, young people, and adults) experience peer governance?
- How do they handle and settle conflicts?

## 3. Media education for civic engagement

### 3.1. Defining the field

As we shall see, the Italian case studies developed the paradigm of the educational commons in conjunction with insights derived from the field of media education and the notion of “participatory culture” (Jenkins, 2006; Jenkins et al., 2009, 2013).

Over the years, the field of media education has developed worldwide as a field of research and educational practice, drawing its epistemological and methodological framework from a variety of disciplines ranging from pedagogy, media studies, sociology of education, psychology, cognitive science, and political science (Kubey, 1997; Christ and Potter, 1998; Buckingham, 2003; Potter, 2004, 2013; Hobbs et al., 2019; Wuyckens et al., 2022).

A fundamental definition inspiring this development was proposed in the early 90s by the US National Association of Media Literacy Education (NAMLE): media education is the process by which (young) people become “media literate” and acquire the ability to access, analyze, evaluate, and create messages in a variety of forms (Aufderheide, 1993). Therefore, although media literacy and media education are often used interchangeably, the former refers to the abilities manifested in the observable actions and practices of media users, and the latter refers to the educational process activated—formally, informally, or non-formally—to achieve them. Users' media practices are interpreted as indicators or “markers” to assess the presence of media literacy skills and, in this case, evaluate the efficiency of media education activities. As with the notion of literacy, it is a question of learning to read and write, a question that has historically determined a conflict between the élites and the masses to the extent that reading is about reading “the word and the world” (Freire and Macedo, 1987) and writing has regulated democratic access to social power (Kress, 1998). A similar conflict has occurred in the field of media education and the opportunity to teach young people to read the media more critically and express their views through their own media productions. Indeed, the critical thrust of media education has often been scaled down in public discourse to merely instrumental and individual skills, diluting its political and emancipatory potential for collective action and civic engagement in the public arena.

The four components identified in the NAMLE definition—access, analysis, evaluation, and content creation—feed into one another within a dynamic educational process. For example, learning to analyze professionally-made media texts helps to create media messages knowing what kind of pragmatic effects they may produce (Hobbs, 2017); similarly, skills in analysis and evaluation contribute to a better understanding of the broader context of media industries or ideology and stereotyping. Indeed, as Buckingham (2007, p. 49) argues, analytical skills can be gained “not only through critical analysis: they can also be developed—in some instances, more effectively and enjoyably—through the experience of creative production.”

The combination of critical thinking with creative production marks a shift from protectionist and moral panic views “against” the media to a more proactive approach, considering the acquisition of media literacy skills as a form of empowerment to help young people benefit at their best from their daily investments in what Jenkins et al. (2009) call “participatory culture.” Echoing the notion of digital commons, participatory culture is a culture “with relatively low barriers to artistic expression and civic engagement, strong support for creating and sharing creations with others, some type of informal mentorship… [and] some degree of social connection with one another” (Jenkins et al., 2009, p. 5–6). The typical practices that young people activate, with different levels of awareness and sophistication, have to do with creating various forms of affiliation, creative expression, collaboration, problem-solving, and circulation in the online environment. Contrary to a naïve view of young people as “digital natives,” naturally endowed with the skills to embrace the participatory culture, scholars, and practitioners from the media education field argue that educators, in combination with public and regulatory institutions and the media industry itself, have a significant role in making sure that young people have access to the skills and experiences needed to become full and responsible participants with a clear understanding of how media shape their lifestyles and worldviews and what ethical standards they should respect in their daily practices as media content producers.

At the policy level, EU institutions have adopted and expanded over the years NAMLE basic definition contributing to consolidating the field in educational settings, both formal and non-formal. The first document is a communication from the EU Commission issued in 2007 and titled “*A European Approach to Media Literacy in the Digital Environment,”* where media literacy is defined as “the ability to access the media, to understand and to critically evaluate different aspects of the media and media contents, and to create communications in a variety of contexts” (European Commission, 2007). The 2016 European Council Conclusions on *Developing Media Literacy and Critical Thinking Through Education and Training* express a more mature view, enriched by the research work and educational initiatives developed over a decade. However, a civic engagement component is added as media literacy is now defined as “closely related to active engagement in democratic life, to citizenship, and the ability to exercise judgment critically and independently, as well as to reflect on one's actions, and can thereby enhance young people's resilience in the face of extremist messages and disinformation.” Moreover, building on a subsidiarity basis, the conclusion states that including media literacy within “comprehensive “whole school” approaches involving the entire school community and other relevant stakeholders can be of great importance since learning to use the Internet, and social media responsibly often takes place outside the classroom in formal and informal settings.” Dialog and cooperation between parents and other stakeholders, such as youth organizations or the media sector, are promoted, “given that the effective development of media literacy and critical thinking calls for a multidisciplinary approach and recalling the important role that formal and informal learning can play in this regard” (European Council, 2016).

Finally, an important mention of media literacy is made in the 2018 EU *Audiovisual Media Services Directive*, the founding policy document by which the EU Parliament and Council guide the Commission and member states in regulating the new “changing market realities” (such as video-sharing platforms). Confirming earlier definitions, the Directive points out that “media literacy should not be limited to learning about tools and technologies but should aim to equip citizens with the critical thinking skills required to exercise judgment, analyze complex realities, and recognize the difference between opinion and fact.” Unlike other documents, however, it adopts a holistic approach where a more substantial involvement of public institutions and the media industry must complement media literacy initiatives. Thus, it invites member states to “ensure that all video-sharing platform providers under their jurisdiction apply a series of measures,” such as, for example, the inclusion of a function for users who upload user-generated videos to declare whether such videos contain commercial audiovisual communications; or transparent and user-friendly mechanisms for users to report or flag to the platform provider content that promotes hate, violence, disinformation, and others to explain to them what effects are produced by their reporting and flagging (European Parliament Council of the European Union, 2018). Indeed, education can play a significant role in teaching people ways to cope with the demands and challenges of the contemporary media environment; however, it is not sufficient. The Directive clearly suggests that it is not only a matter of individual responsibility to become “media literate,” as Buckingham (2019) argues, “Media is not a substitute for media regulation […]. We need a much more comprehensive approach to understanding how media might be mobilized in the interests of the public good. In the process, we need to recognize the limitations of what education itself can achieve […] Educators need to work with other public and non-governmental bodies in seeking to promote wider changes and reforms” (p.115–116).

### 3.2. Overcoming the “civic gap”

Although the conceptual definition of media education as a process to raise critical awareness has been widely explored in media education research, much less has been explored about how this should translate into civic engagement in everyday life (Banaji and Buckingham, 2013; Mihailidis, 2018, 2019).

In this regard, Boyte (2014) discusses a concept called the “civic gap,” which refers to the difference between being aware of a problem and being able (or willing) to act to address it. Due to this gap, media education, while confirming its status as an educational practice aimed to train critical analysis skills and creative production of media texts, is facing new challenges to (re)affirm its “civic intentionality,” i.e., “a set of design considerations for media literacy initiatives that are based on the value systems of *agency, caring, persistence, critical consciousness, and emancipation*” (Mihailidis, 2018: p. 2, italics in original). Through such intentionality, young people will put forth a positive dialog in their community and a “sense of being in the world with others toward the common good” (Gordon and Mihailidis, 2016, p. 2).

**Figure 1:**
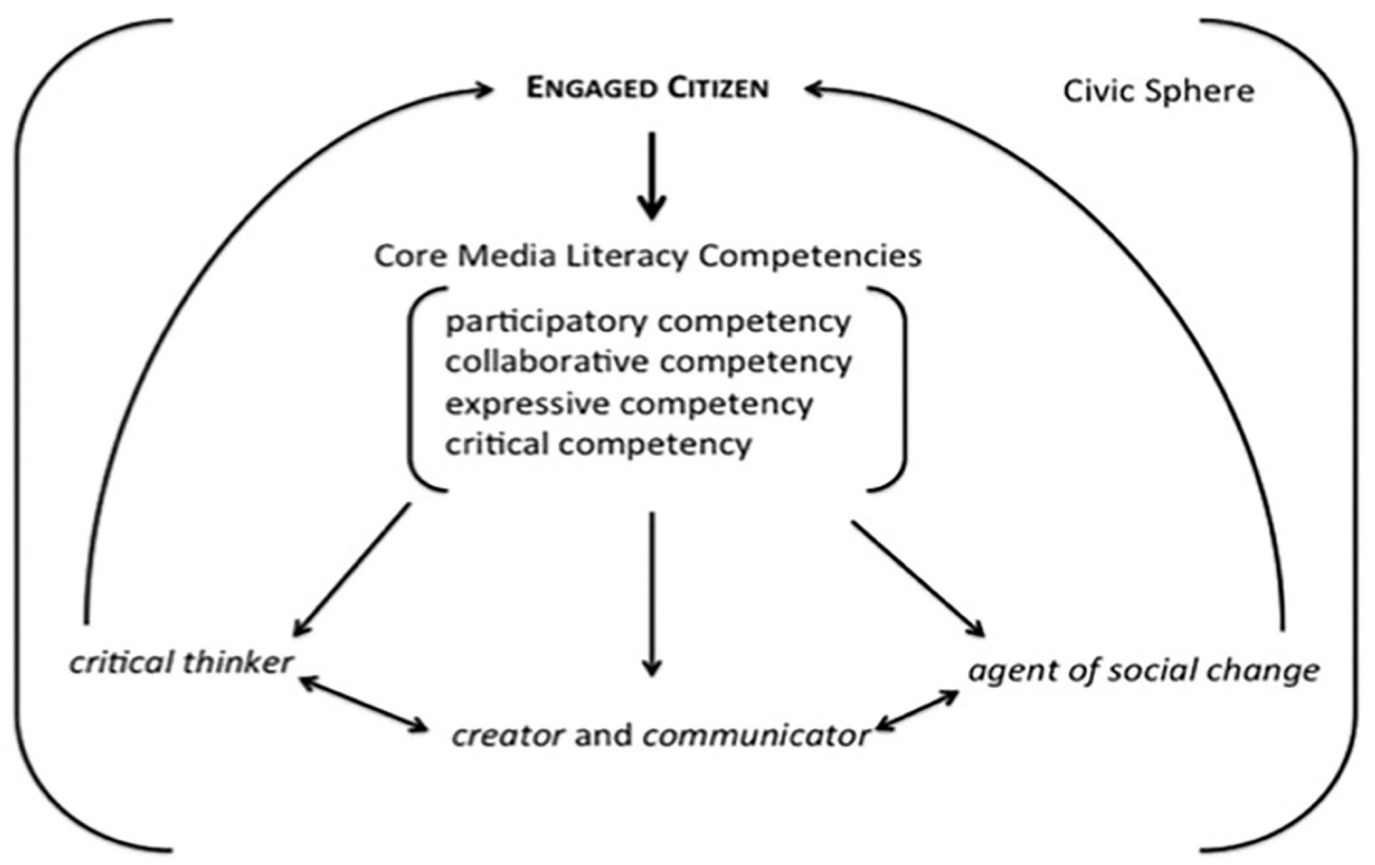
A framework for media literacy as a core competency for engaged citizenship. Source: Mihailidis and Thevenin (2013, p. 1,617).

To fill the civic gap and (re)construct this civic intentionality, Mihailidis and Thevenin (2013) argued that the traditional skills of critical analysis and creative production are to be placed within a more general framework of civic values (see Figure 1).

Mihailidis' and Thevenin's model is centered around four “core media literacy competencies” that every engaged citizen should develop. The first two, participatory and collaborative competency, focus on the notion of participatory culture and imply the acquisition of skills that “make it possible for average consumers to archive, annotate, appropriate, and recirculate media content in powerful new ways” (Jenkins et al., 2009, p. 8). However, participatory and collaborative media literacy competencies operate at the macro level of engagement, and expressive media literacy competencies focus on the content that each young citizen creates and shares at a personal level but with a possible resonance at the public level too: “By focusing on the creation, dissemination, and reception of individual expression, young citizens can reflect on the content of their voice and also on the power they have to be part of a larger civic dialogue” (Mihailidis and Thevenin, 2013, p. 1,618). By acquiring critical skills, the engaged citizen takes a critical distance from media messages and ponders their ideological and commercial implications.

The implementation of this framework largely depends on the conditions and possibilities within which social actors (students, schools, families, NGOs, public authorities, and policymakers) operate locally. In any case, as Mihalidis and Thevenin concluded, “while there may be no single metric or normative position for a “good citizen,” it seems that in an increasingly mediated world, citizens with the capacities to participate, collaborate, and express themselves online stand a better chance to become *critical thinkers, creators, communicators, and agents of social change*” Mihailidis and Thevenin (2013, p. 1,619, italics in original).

## 4. The Italian case studies of the SMOOTH project

### 4.1. A general description

The two Italian case studies (CSs) involved the same target group (young people aged 12 and 16 years) and were both implemented in two non-formal educational contexts with some similarities but also some specific characteristics. Both contexts are youth clubs that aim to promote initiatives using a bottom-up approach. One CS is taking place at the Centro Tau (http://mediatau.it/centrotau/), located in the La Zisa neighborhood, one of the most at-risk areas of the metropolitan city of Palermo, characterized by high rates of job insecurity and unemployment, early school leaving, child labor, and delinquency. Centro Tau was established in 1990 by *Inventare Insieme*, a non-profit organization that offers children and young people in La Zisa a wide range of educational and cultural opportunities. The other CS is in Agrigento, in a youth club run by *Caritas-Fondazione Mondo Altro* (https://www.caritasagrigento.it/fondazione-mondoaltro/), a Catholic organization addressing the needs of disadvantaged people in the local community through innovative social and educational initiatives. Its areas of intervention include migration, disability, voluntary work, international cooperation, and support for poverty. The young people involved in the CS are primarily from immigrant backgrounds.

The collaboration with Centro Tau and Caritas is based on the idea that local networks and the exchange of multidisciplinary skills and practices between formal and non-formal educational contexts are crucial for engendering change in the community, regardless of the highly disadvantaged conditions that characterize it.

As anticipated in the previous paragraphs, our CSs developed the notion of educational commons in conjunction with insights derived from media education and the notion of participatory culture, adopting an action-research approach. It is well-known that the Internet, and more recently, social media, allow the formation of new modes of production and collaboration that pave the way to novel patterns of association and self-governance. Grafting media education into this participatory culture encourages youths to develop the skills, knowledge, ethical and critical frameworks, and self-confidence needed to be fully “engaged citizens.”

Building on this framework, we articulated our activities for the CSs in three phases:

1. *Training of the educators* (February/April 2022). We organized a 30-h training on “photography and social media,” framing it within an educational-commons and media-education approach. Co-creation, peer-to-peer education, and action research were part of the training to better take into account the contexts as well as the specific needs and desires of the people (educators and young people) involved in the CSs;
2. *The first round of implementation of the CSs* (May/September 2022). After the training, educators started working with young people to develop their “core media literacy competencies.” Together with activities of critical analysis of images, young people were asked to take pictures, individually and in a group, either at the youth club or during outdoor walks in the neighborhood. As planned, a blog was also created where young people could upload these pictures, comment on them, and share them to introduce to the general public their views and opinions on various issues concerning their personal lives or the local community they live in. Indeed, since photography and social media are essential components of young people's daily lives, these activities can be highly motivating for learning to engage in media practices in a more critical, creative, responsible, and pro-social way;
3. After the first analysis of the preliminary findings from the first round, the second round of implementation is planned (January/June 2023), including all possible changes and improvements derived from the analysis.

After the second round, through the content co-created with young people and shared on the blog, we plan to engage the local community with the issues chosen by the young people. These productions will be exhibited in offline contexts so that young people can interact publicly with other people through face-to-face encounters. The active involvement throughout the experimentation of all the people directly and indirectly involved in the CSs (organizations' leaders, educators, young people, and families) will undoubtedly contribute to constant monitoring and assessment of the CS.

### 4.2. The training phase

Training is central to developing “civic intentionality” in young people and the “commoners” around them. By gaining such “intentionality,” they will build a positive dialog with their community and a strong sense of belonging based on sharing a process of growth, development, and reciprocal education.

The training follows a cascade development: in the first round, it goes from university researchers to educators and then from them to the young people; in the second round, the young people will train their peers and, if possible, their parents.

As mentioned, the educators received training in photography and social media, raising their awareness of the associated risks and opportunities. Some of the training sessions were dedicated to co-designing an implementation plan for the activities with the young people involved in the co-creation activities after being trained by the educators. This plan, like the training provided to the educators, was divided into five modules. The first module aimed to acquire the basic visual literacy skills needed to take photographs with a mobile phone. The use of the mobile phone was preferred to digital cameras as we wanted to engage young people with media tools and practices with which they were already familiar. The second module aimed to create a photograph blog where young people could publish, comment on, and share their co-produced content. The last three modules were dedicated to practical exercises to acquire more specific skills. In particular, the third module was dedicated to developing skills in selecting the subject, the moment, and the location, making young people aware of the complex composition of an image. The fourth module was dedicated to interpretation. In this case, young people learn how to interpret images, how they create meaning, and how to identify critical elements in the meaning-making process. The last module focused on storytelling, i.e., the ability to narrate and communicate through images, select pictures to build a story, choose a “rhythm” (from start to climax and conclusion), and find a pragmatic coherence between intention and effect.

In Agrigento, the training took place between February and April 2022 and involved a male educator working full-time for Caritas and a small number of temporary volunteers. In Palermo, it took place in June 2022 and involved eight educators (six women and two men) working full-time at the Centro Tau.

## 5. The fieldwork research: Participants, tools, and analysis dimensions

We are in the process of analyzing the vast amount of data collected in the first round of implementation of the CSs. In this study, we report on the fieldwork and discuss some preliminary findings from the implementation of the CS carried out in the *Centro Caritas* of Agrigento.

Participants were one male educator and 10 women (five girls and five boys) aged 12–14 years.

**Figure 2:**
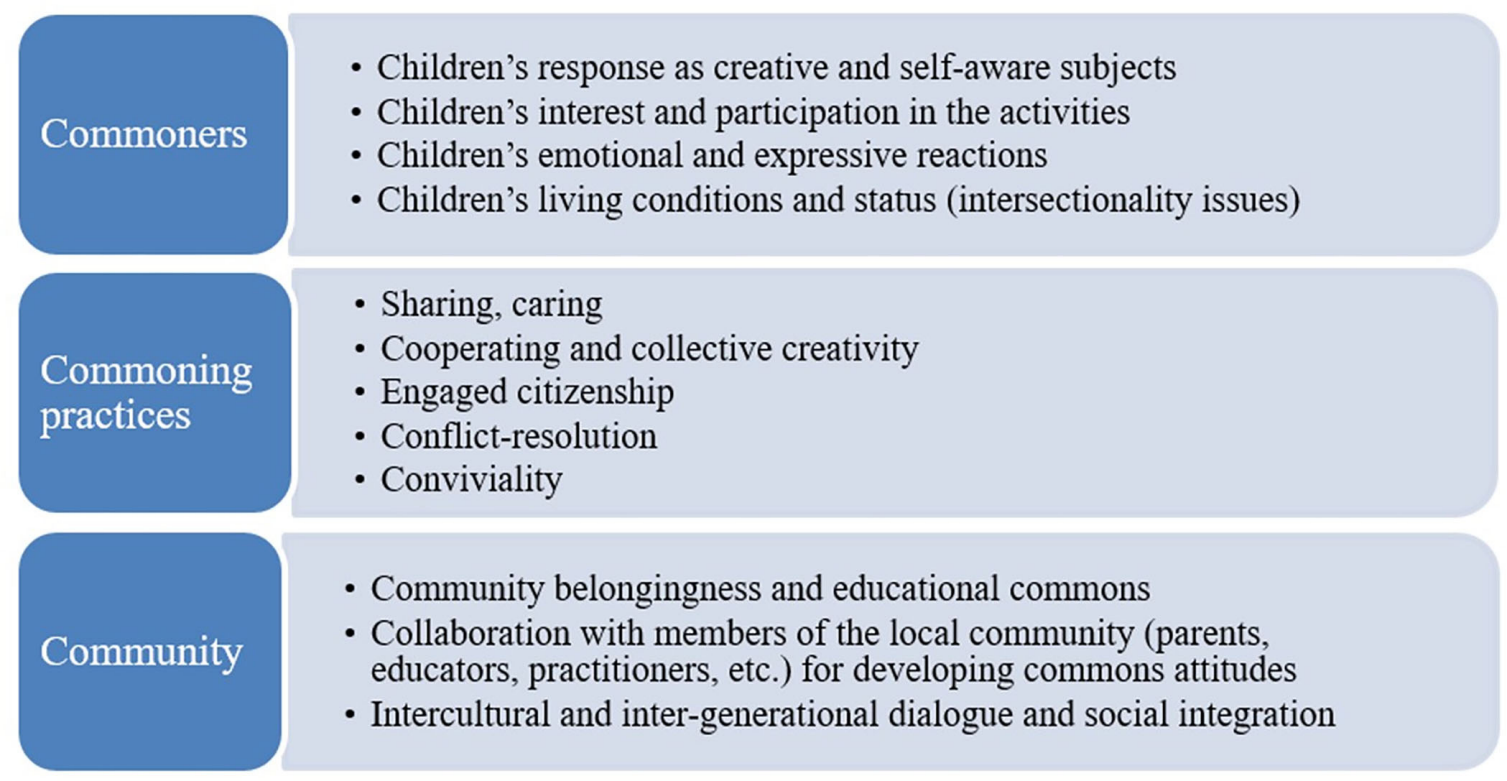
Core research dimensions of the case study.

Fieldwork research is an essential part of CS implementation. Its primary purpose was to assess the impact of the activities, considering the three core research dimensions of the educational commons identified in the SMOOTH theoretical framework (see Figure 2). We used a qualitative approach, focusing on the visual as a narrative element. Data were collected before, during, and after the round of implementation. Fieldwork involved ethnographic research tools (participant observation, focus groups, in-depth interviews, audiovisual recordings, photographs, drawings, and so on).

The ex-ante data collection investigated perceptions and expectations on the SMOOTH project's main topics, setting the basis for co-creating and starting a shared learning process. It was carried out in March 2022 by university researchers and included (a) the compilation of a participant's profile form to collect information such as gender, age, family composition, and so on); (b) one focus group with the educators involved in the training; (c) one in-depth interview with the *Centro* manager; (d) one interview with the educator supervising the CS implementation; and (e) one focus group with the young people involved.

The focus groups with the educators and the young people were fundamental to co-designing the activities and adapting them to the specificities of the context and the needs/desires of the participants. At the start of the activities, we also planned a focus group with parents. However, we could not involve them due to their suspicion of activities with new “figures” (university researchers) from outside the *Centro*. We plan to contact them again in the intermediate phase between the two rounds to check whether they have changed their mind after seeing their children actively involved in the CS. If they have, we plan to jointly decide with them how they can be part of the second round, starting in January 2023.

The constant monitoring of the CS activities during the first round (May/September 2022) actively involved university researchers, educators, and young people, in line with the participatory perspective of action research. The same will occur during the second round (January-June 2023).

The collection tools used during the activities in the *Centro Caritas* were:

- A collective logbook for youth, i.e., a flipchart where young people wrote for each session a few words about what they did and learned. Photographs could also be added.
- The educator's logbook with fieldnotes (both descriptive and more subjective, including impressions, emotions, and perceptions);
- The researcher's logbook with fieldnotes (both descriptive and more subjective, including impressions, emotions, and perceptions);
- The researcher's observation grid;
- A GANTT chart of the activities;
- An activity documentation sheet (compiled by the educators and the researcher) describing every single activity carried out (content and purpose, number of participants, places and times, implementation methods, and possible deviations from the plan), the learning context (participation of young people, group management, and conflict resolution), the achievement of objectives (overall judgment, critical points, and solutions, and the lesson learned). Photographic and audiovisual material is part of the documentation.

Therefore, the empirical basis of our analysis is made up of the textual data collected through the interviews and focus groups, the logbooks, the field notes, the observation grids, and the written and audiovisual documentation. By building on these data and comparing findings from the two different rounds per CS and from one context (Agrigento) to the other (Palermo), it will be possible to (a) reconstruct the micro-context in which the activities took place, (b) comprehensively describe the relational dynamics using unobtrusive methods, the processes, and the products, and (c) assess the impact of the activities implemented considering the double objectives of developing “civic intentionality” and reducing educational inequalities through an educational-commons and media-education approach. The analysis will be guided by the application of the three core dimensions of the SMOOTH project (see Figure 2). Each dimension is further specified into sub-dimensions, with specific research questions co-formulated and validated with the educators during the training done before the start of the first round of activities.

The research plan also envisages an ex-post survey carried out at the end of the two rounds (October/November 2022 and June/July 2023) by university researchers with the aim of evaluating the whole process, from start to finish, of the CS. This phase includes the same data collection plan as the ex-ante phase so that results can be compared. In the ex-post data collection phase scheduled between the two rounds, particular attention will be paid to the co-design of the second round, using, if necessary, the evidence that emerged from the first round.

## 6. Preliminary findings

Following the three core research dimensions of the SMOOTH project (see Figure 2), we present here a preliminary and exploratory analysis of some of the findings that emerged from the first experimental round carried out in Agrigento from May to September 2022. Activities were carried out during a total of 7 sessions. In each session, data were collected through two logbooks (one compiled by the educator and the other by a researcher-observer). Each logbook details what occurred during each session and included a series of comments and reflections. In addition, we gathered data by compiling an observation grid for each session (a total of seven grids) and a documentation sheet for the activities performed in a single session.

### 6.1. Commoners

The commoners' dimension was investigated and analyzed through four sub-dimensions regarding young people in particular: (a) their response as creative and self-aware subjects; (b) their interest and participation in the activities; (c) their emotional and expressive reactions; and (d) their living conditions and status (intersectionality issues).

The specific research questions for this dimension were as follows:

- What interested most young people?
- How did they express this interest: with gestures, words, glances…?
- What were they trying to understand: which questions and problems were they formulating?
- What differences and similarities can we see in interests, expressions, questions, and problems?
- How and when did young people express joy at doing things individually and collectively?
- Did intersectional issues (socio-economic status, migratory background, disability, and so on) affect young people during the activities? If yes, who, when, and how?
- Were they aware of what they were doing and why?

Young people enthusiastically participated in the activities but were not consistent either within the same activity or across them. As one educator writes in the logbook, “It's difficult to keep their attention, but ultimately they completed their tasks!” emphasizing this as an achievement in itself, given the typical inconstancy of adolescents. They showed a strong interest in new activities and preferred to work in a group rather than individually. They also showed good expressive competence: They explicitly manifested their approval or disapproval of the activities, sometimes even walking away. They were also quite creative on several occasions and gradually increased their awareness of what they were doing, i.e., they focused more on the assigned topic and the general aims of the activities. Finally, intersectionality was a key challenge, mainly because different ethnic groups worked together (some were natives and others had a migrant background). This condition is unique to the local context of Agrigento and must be considered when designing the second round's activities.

### 6.2. Commoning practices

The sub-dimensions analyzed for this dimension were: (a) sharing and caring; (b) cooperating and collective creativity; (c) engaged citizenship; (d) conflict resolution; and (e) conviviality.

This dimension investigated the relational dynamics within the group to capture the change concerning the existence and frequency of pro-social and cooperative behavior and the development of conflict resolution skills. In particular, we focused on how the boundaries between what is considered “mine” and “yours” were negotiated between the participants and on the recognition of a collective dimension in which to invest. Therefore, the specific research questions of this dimension were:

- How were youth empowered as social agents actively involved in public life (i.e., youth as commoners, active users, and co-creators of educational commons)?
- How did they discover and foster their own priorities and improve active inclusion?
- How were they involved and engaged in individual and collective activities?
- Did they show care and concern about themselves and each other?
- How did they express feelings about themselves and others by being able to act, think, and talk freely in public?
- How did they develop skills for peer governance, shared rules, rights, obligations, and decision-making?
- What kind of attitude/behavior did they adopt to manage conflicts in group activities?

As young people were already part of a community (the youth club) that typically develops participatory methods, they were used to working in groups and sharing everything they used during the activities. However, they were unaware that what they co-produced belonged to everyone. They do things together and share tools and experiences, but they need more awareness that an action can have a shared aim and produce a co-created experience with possible effects for the whole community. This awareness emerged mainly during the last sessions of the first round but needs to be further consolidated during the second. Young people have become more and more aware that they can contribute to group activities in an active way. However, they do not yet consider it achievable or even desirable that their engagement can be valuable for the whole local community. Their agency is therefore limited to everyday personal activities and their micro-context of living (both at home and at the *Centro*). The photograph blog experience (albeit still in its initial phase) represents, in this sense, an interesting example of an activity (a “commons”) that may go beyond the social relations developed within the peer group or the family. As we expect to verify with the second round, it may become a space for experimenting with practices that may have an impact at the community level.

Conflicts (arising in most cases over trivial matters) always required the educator's presence to help the group in resolving them. Some of the young people showed good mediation skills and actively contributed to conflict resolution when encouraged by the educator.

The cooperation between young people and educators was excellent, except in a few cases where conflicts were nonetheless resolved. One episode is worth mentioning. During a session, one of the boys appeared distracted, and the educator invited him to pay more attention, reminding him that staying until the end of the meeting was not mandatory. When he repeated this a bit later in this invitation, the boy just left without a word of explanation, clearly “offended” by the educator's words. What happened immediately afterward is also worth noting: for the following meetings, half of the boys no longer went to the club as they had been “forbidden” by the offended boy. Fortunately, with much effort, the educator managed to take them back little by little. Even the offended boy, whom the others called “pack leader,” began to follow the meetings again. In the end, although almost the whole group had been affected by this sudden conflict, the group managed to solve it and reinforce cooperation and a sense of group belonging.

### 6.3. Community and the commons goods

The sub-dimensions analyzed for this dimension were (a) community belongingness and educational commons; (b) collaboration with members of the local community (parents, educators, practitioners, and so on) to develop commons attitudes; and (c) intercultural and intergenerational dialog and social inclusion.

This dimension investigates the macro level, the broader context that includes commoners (young people and adults) and common practices. Individual actions may have an impact at a collective level and acquire a value that goes beyond the individual perspective. In this case, the analysis looks at the “educating community” attempting to define its function and liminal borders. The research questions specific to this third dimension were:

- How did the embeddedness in the local context of the CS contribute to developing educational commons and a sense of community belonging?
- How did the CS demonstrate that education can be effectively organized based on common patterns?
- What were the effects at the community level of applying the commons' logic to address inequalities and intersectionality issues and achieve social inclusion of youth from vulnerable social groups?
- Can commons-based peer education contribute to the further development of commons-based peer production? What kinds of “products” (routines, activities, tools, artifacts, and more) were generated by the commoners in the commoning processes? And how does this impact the broader community?
- How did commoners experience peer governance?

The young people showed a good attitude toward dialog and respect for others (even those different from them in terms of ethnicity, origin, age, and so on). They recognize the club as a community they feel they are members of. However, outside the center, the different “memberships” remain clearly marked.

The activities strengthened the young people's ties within the club's community, triggering mutual recognition. They also timidly initiated a process of opening toward the world outside the club, discussing the impact of their actions on the local community. The photograph blog has great potential for that. We expect to develop and consolidate this potential during the second round, making it a space where actions in a micro-context like a youth club can be visible and possibly produce some effects on the broader macro-context. Parents and the local community have not yet been engaged in the activities. We plan to do that in the second round.

## 7. Discussion and conclusions

This study is indeed a work in progress. It describes the general action-research framework adopted in the experimentation of the Italian CSs of the project SMOOTH, highlighting the circularity between design, action, and re-design. Fieldwork was developed by investigating the three main dimensions of the notion of educational commons. They were first theoretically defined and then operationalized in less abstract sub-dimensions to empirically detect helpful information to assess the impact of the intervention. The ambition of the SMOOTH project, particularly for our CSs, is to investigate: (a) how young people collectively experience and construct the commons in educational settings; (b) how young people and adults in the educational community experience peer governance and how they manage and resolve conflicts within the community they belong to; (c) how the co-creation of a photograph blog as a shared online space of work helps young people discover and develop civic intentionality not only online in the digital environment but also in offline contexts; and (d) what the effects of applying a commons' logic to address inequalities and achieve social inclusion are for young people from vulnerable social groups.

Given the data collected so far, neither do we have detailed answers to these questions nor can we argue that the “civic gap” has been overcome. However, we can undoubtedly make some preliminary reflections on what worked well in the first round and what could hinder the achievement of the project's objectives. We can also propose hypotheses for re-designing the second round to overcome the weaknesses that emerged during the first experimental phase and enhance its strengths.

Indeed, the bottom-up approach adopted in the youth club of Agrigento was a strength. The educators were already familiar with participatory methods and used them in their daily work with young people. However, during the CS activities, they learned to value the theoretical framework within which they were asked to apply them: a community of commoners who share a commonality: education. The same thing seems to be happening among young people: they feel free to propose, to create, to imagine paths, and to express their needs, desires, and also disapproval.

Academic researchers were also part of this common experience. We had to adapt our initial training plan to the characteristics and requests coming from the educators. Together with them, we designed the implementation plan for the CS, and the educators, in turn, adapted it to the young people and, from time to time, to the specific needs and desires of individual group members. Relations were constantly based on active listening, paying attention to the ever-changing demands and needs of all community members. Those who could not do so at the beginning of the experiment gradually learned to do it.

These aspects must be further investigated and confirmed during the second round. Constant monitoring and assessment are fundamental to the success of our research and re-designing activities.

Admittedly, some dysfunctional elements revealed shortcomings and represent a challenge we are currently reflecting on. The first critical element regards the awareness-raising process and how it was affected by the emotional fragility typical of adolescence, a period when children go through a real emotional storm in search of their own individual and social identity and ways forward to their future. Day by day, they discovered themselves and the world around them, slowly and painstakingly building their own personal and social imagination. During the elusive and ephemeral phase of their life path, it is rather complex to structure a process of conscientization, as Freire (1970) would put it, by introducing new values and raising awareness of social issues that, at this time, may be perceived as “distant, external, and adult-like.”

A second major issue was the inconstancy and unpredictability of young people's attendance at the round sessions (as the episode mentioned in Section 6.2 shows). It is not easy to form a group of adolescents and keep it constant in its composition until the end of the activities. In Agrigento, we managed to achieve this with the crucial support of the educator. Nonetheless, attendance was an issue: young people did not attend some sessions, and attention during the activities was not constant. We know well that this may also be caused by the fact that in non-formal educational contexts, unlike formal ones, attendance is not mandatory and is mainly determined by the interest that activities can generate in children. The fact remains that inconsistent attendance may be problematic in a process aiming at the progressive acquisition of competencies where active participation is required and not simply mere involvement. We also know that inconstancy could be a problem in maintaining a regular collective flow of publications on the photograph blog during the second round. We hope that the need to have a constant flow of publications may trigger a better use of time and make attendance and engagement more constant and regular.

The last but not the least important issue to consider is the outbreak of conflicts (fortunately, always temporary and not severe) within the group due to the controversial nature of peer and adult-child relationships at this age. Findings seem to suggest that conflicts can be resolved, but this cannot be taken for granted, nor can group cohesion. The conflict episode recalled in Section 6.2 indicates that, during the second round, we need to pay close attention to some important criticalities: the difference between being a leader and a “pack leader,” the risks of imitative dynamics, the gender dimension, the importance of supporting the pursuit of a collective goal (the group activities planned for the CS) over personal interest and feelings (the “offense”), the need to reiterate the value of dialog and confrontation as an alternative to conflict (or walking away in the case of the boy leaving the meeting).

All these critical issues need to be carefully considered in the intermediate data collection, during which educators and young people will propose solutions and implement new activities to overcome these criticalities. The first round was a valuable experiment to prepare the context for a more structured intervention that fully uses the educational commons approach. The second round will extend the first, further developing commoning processes. The young people trained during the first round will, in turn, become trainers of new young people. We also aim to involve parents, schools, and the local community in the commoning and media education process of “reading and writing” a reality using media language. We aim to foster in all participants both a “sense of being in the world with others toward common good” (Gordon and Mihailidis, 2016, p. 2) and a process of “agency, caring, persistence, critical consciousness, and emancipation” (Mihailidis, 2018, p. 2).

## Data availability statement

The datasets presented in this article are not readily available because the dataset is not available to the general public. Requests to access the datasets should be directed to gianna.cappello@unipa.it.

## Ethics statement

The studies involving human participants were reviewed and approved by Prof. Vito Di Marco, Prof. Marianna Alesi, Prof. Alice Pugliese, Prof. Marco Brigaglia, and Prof. Pietro Perconti, University of Palermo. Written informed consent to participate in this study was provided by the participants' legal guardian/next of kin.

## Author contributions

GC wrote Sections 2, 4, and 6. MS wrote Sections 3, 5, and 7. The introduction (Section 1) was written by GC and MS. All authors contributed to the article and approved the submitted version.

## Footnotes

^1^Action-research is widely used in media education research. For example, see: Buckingham and Sefton-Green (1996), Ranieri (2016, 2019), and Bruinenberg et al. (2021).
